# Trans-Arterial Chemoembolization with 50 μm Degradable Starch Microspheres Versus 300–500 μm Drug Eluting Beads in Hepatocellular Carcinoma: A Comparative Analysis of Initial Treatment Outcomes

**DOI:** 10.5334/jbsr.2594

**Published:** 2022-03-10

**Authors:** Isil Yildiz, Sinan Deniz, Ali Ozer, Kosti Caliskan

**Affiliations:** 1 Acibadem University Atakent Hospital, TR; 2 LMU Klinikum Grosshadern, DE

**Keywords:** hepatocelluler carsinoma, trans-arterial chemoembolization, degradable starch microspheres, drug eluting beads, treatment outcome

## Abstract

**Background and Aims:**

Trans-arterial chemoembolization (TACE) has become a widely accepted treatment in unresectable hepatocellular carcinoma (HCC). We aimed at comparing the efficacy of Degradable Starch Microspheres (DSMs)-TACE with 50 ± 7 *µm* versus 300–500 μm Drug Eluting Beads (DEB)-TACE in terms of initial clinical and radiological treatment response parameters.

**Material and Methods:**

A total of 54 patients with unresectable HCC who underwent DEB-TACE (n = 25) or DSMs-TACE (n = 29) were included in this retrospective study. Baseline demographic and clinical characteristics, duration of follow-up, local recurrence and survival status, as well as treatment outcome including treatment response via modified Response Evaluation Criteria in Solid Tumors (mRECIST) criteria, viable and total tumor diameter and serum alpha-fetoprotein (AFP) levels were analyzed in both study groups.

**Results:**

No significant difference was noted between the two groups in terms of local recurrence (31.6 vs. 16.7%) or mortality (73.9 vs. 85.7%) rates after 36-month and 12-month follow-up, respectively. DSMs-TACE vs. DEB-TACE was associated with significantly higher complete response rate (27.6 vs. 0.0%, p = 0.011) and significant decrease in serum AFP levels (p = 0.013).

**Conclusion:**

Both DSMs-TACE with 50 ± 7 *µm* microspheres and 300–500 μm DEB-TACE are effective for local control of unresectable HCC. Our findings revealed superiority of DSMs-TACE over DEB-TACEnin terms of initial clinical and radiological tumor response; though no significant difference was noted between the two patient groups in terms of local recurrence or mortality during follow up.

## Introduction

Trans-arterial chemoembolization (TACE) has become a widely accepted treatment in unresectable HCC patients with a relatively preserved liver function [1,2].

However, both conventional-TACE (c-TACE) and drug eluting beads (DEB)-TACE have been shown to have high rates of tumor recurrence, possibly due to vascular-endothelial-growth-factor (VEGF)-mediated new tumor vessel formation following extended ischemia in surrounding normal liver [3,4,5,6].

In this regard, the use of degradable starch microspheres (DSMs) for transient occlusion of tumor feeding arteries has been proposed as an alternative TACE method to avoid VEGF overexpression [7,8].

However, in addition to scarce number of studies on the efficacy and safety of DSMs-TACE among patients with unresectable HCC [8,9,10,11,12,13], no study to date has compared the clinical utility and efficacy of DSMs-TACE and DEB-TACE in HCC patients.

This retrospective single-center study was therefore designed to compare the efficacy of DSMs-TACE with 50 ± 7 *µm* microspheres versus DEB-TACE with 300–500 μm caliber beads in unresectable HCC patients in terms of clinical and radiological treatment response parameters.

## Material and Methods

### Study population

A total of 54 consecutive patients with unresectable HCC who underwent doxorubicin-based TACE via DEB (DEB-TACE, 300–500 μm, n = 25) or DSMs (DSMs-TACE, n = 29) in accordance with the decision of the multidisciplinary team of specialists were included in this retrospective study conducted between May 2016 and October 2018.

Patients with the histopathological diagnosis of HCC based on American Association for the Study of the Liver Disease (AASLD) guidelines, receiving DEB-TACE or DSMs-TACE, no treatment prior to or during the intervention, availability of data on pre-TACE and post-TACE 1-month assessments, Eastern Cooperative Oncology Group (ECOG) performance status of 0–1, Child-Pugh Class A–B, normal renal, cardiac and bone marrow functions, total bilirubin levels < 3 mg/dL, albumin levels > 2.0 mg/dL and at least 12 weeks of life expectancy were included in the study. The presence of an extrahepatic metastasis, tumor volume over 70% of total liver volume, uncontrolled hypertension, bleeding diathesis, secondary malignancy, Child-Pugh Class of C, combined treatment or treatment with 100–300 μm DEB and previous anti-cancer treatment were the exclusion criteria of the study.

Written informed consent was obtained from each subject following a detailed explanation of the objectives and protocol of the study which was conducted in accordance with the ethical principles stated in the ‘Declaration of Helsinki’ and approved by the institutional ethics committee.

### Study parameters

Data on baseline characteristics including age, gender, comorbidities, Model for End Stage-Disease (MELD) scores, compatibility with Milan criteria, Barcelona Clinic Liver Cancer (BCLC) stage, positron emission tomography (PET)-Computed Tomography (CT), presence of macrovascular invasion, and transplantation were recorded in each patient. Data on follow-up duration (months), local recurrence and survival status, as well as treatment outcome including treatment response via modified Response Evaluation Criteria in Solid Tumors (mRECIST) criteria, viable and total tumor diameter (diameter of the lesion in case of one lesion, total diameter in case of more than one lesions) and serum alpha-fetoprotein (AFP) levels were recorded in study groups.

### Treatment outcome

Treatment outcome was evaluated in terms of radiological (Magnetic Resonance Imaging [MRI], viable tumor diameter before and after procedure and mRECIST criteria) and clinical (serum AFP levels) response parameters after one month of treatment. Patients were evaluated with contrast enhanced CT or dynamic MRI at time of enrollment and 25–30 days after each DSMs-TACE or DEB-TACE procedure. Radiological evaluation was performed by the same independent seven-year experience abdominal radiologist who was blinded to the treatment protocol of the study. Treatment response evaluation based on m-RECIST criteria was performed and the response was categorized as complete response (CR), partial response (PR), stable disease (SD), and progressive disease (PD) (20). In patients with more than one TACE sessions, assessment included only the first TACE. In patients with multiple tumors, combined assessment of target lesions, nontarget lesions, and new lesions was performed.

### DEB-TACE procedure

The femoral artery access was used in all patients. Angiographic images of the main hepatic artery and superior mesenteric artery were obtained via macrocatheter (USL2, Cordis, USA) and 0.038 hydrophilic wires (Terumo, Japan) to assess anatomy of arteries supplying liver and patency of portal vein. The arteries supplying the tumor were selectively catheterized with a 2.7 F coaxial microcatheter (Progreat, Terumo, Japan) with adapted microwire within a macrocatheter. Through the microcatheter, adriamycin (30 mg/m^2^)-loaded polyvinyl alcohol particles (DcBeads® 300–500 μm; Biocompatibles, Farnham, UK) mixed with iodinated contrast were injected very slowly with fluoroscopy guidance. Each vial contained 50 mg doxorubicin. Injection was continued until near stasis without exceeding the two vial (100 mg doxorubicin). No major complications occurred. After the procedure patients were hospitalized for one or two days depending on the clinical condition.

### DSMs-TACE Procedure

DSMs-TACE was based on the same technique with DEB-TACE, but with use of adriamycin (30 mg/m^2^)-loaded EmboCept® S DSM 35/50 (PharmaCept GmbH, Berlin, Germany), as an embolizate composed of degradable starch microspheres with an average diameter of 50 micrometers.

### Statistical analysis

IBM SPSS Statistics for Windows, version 25.0 (IBM Corp., Armonk, NY) was used in statistical analysis. Pearson Chi-Square test, Fisher Exact Test (Exact) and Fisher Freeman Halton Test (Monte Carlo) were used to analyze categorical data. p < 0.05 was considered statistically significant.

## Results

### Baseline characteristics, duration of follow up and survival status in study groups

DSMs-TACE and DEB-TACE groups were similar in terms of age and gender, MELD scores and comorbidity status.

DEB-TACE group was associated with significantly higher percentage of patients with BCLC Stage-intermediate stage disease (80.0 vs. 41.4%, p = 0.011), and with positive PET imaging (100.0 vs. 79.3%, p = 0.025), while DSMs-TACE group was associated with higher rates of BCLC-early stage (27.6 vs. 4.0%, p = 0.011) and BCLC-advanced stage (31.0 vs. 16.0%, p = 0.011) disease.

Overall, after 29 months (range, 12.5 to 36.5) of follow up, local recurrence was noted in 28.0% of patients and mortality occurred in 42.6% with no significant difference between DSMs-TACE and DEB-TACE groups in terms of local recurrence (31.6 and 16.7%, respectively) or mortality rates (73.9 and 85.7%, respectively).

### Treatment outcomes

Complete response rate was 8/54 (14.8%) in the overall study population, and significantly higher in DSMs-TACE group as compared with DEB-TACE group (27.6 vs. 0.0%, p = 0.011). Overall, 40/54 (74.1%) of patients achieved partial response, with no significant difference in DSMs-TACE (65.5%) and DEB-TACE (84.0%) groups (***Table 1***).

**Table 1:** Treatment outcome in study groups.


	TOTAL (N = 54)	DSMs-TACE (N = 29)	DEB-TACE (N = 25)	P VALUE

**mRECIST criteria, n(%)**				

Complete response	8 (14.8)	8 (27.6)	0 (0.0)	**0.011^ff^**

Partial response	40 (74.1)	19 (65.5)	21 (84.0)	

Stable disease	5 (9.3)	2 (6.9)	3 (12.0)	

Progressive disease ^Excluded^	1 (1.9)	0 (0.0)	1 (4.0)	

**Viable tumor (mm), median (Q1/Q3)**				

Before procedure	40 (31/57)	40 (30/53)	42 (32/60)	0.414 ^u^

After procedure	12 (4/22)	7 (0/15)	18 (9/34)	**0.007 ^u^**

Difference (absolute, mm)	–28 (–38/–19)	–29 (–38/–22)	–22 (–35/–12)	0.423 ^u^

Difference (%)	–70.8 (–90.3/–55.0)	–81.2 (–100.0/–66.7)	–66.7 (–73.3/–46.2)	**0.005 ^u^**

**P-value (intragroup)**	**<0.001 ^w^**	**<0.001 ^w^**	**<0.001 ^w^**	

**Total tumor (mm), median (Q1/Q3)**				

Before procedure	48 (35/70)	45 (38/56)	56 (33/82)	0.253 ^u^

After procedure	34.5 (21/67)	30 (20/35)	50 (25/75)	**0.019 ^u^**

Difference (absolute, mm)	–11.5 (–21/–3)	–15 (–22/–8)	–9 (–15/0)	0.056 ^u^

Difference (%)	–24.7 (–37.5/–6.7)	–32.1 (–45.3/–20.0)	–13.3 (–30.0/0.0)	**0.005 ^u^**

**P-value (intragroup)**	**<0.001 ^w^**	**<0.001 ^w^**	**0.024 ^w^**	

**AFP (ng/mL), median (Q1/Q3)**				

Before procedure	59.85 (11/1546.2)	29.5 (6.8/98.3)	1558 (32.4/11116)	**0.001 ^u^**

After procedure	22.35 (3/478.8)	11.4 (2.3/40)	242 (8.1/6132)	**0.009 ^u^**

Difference (absolute, mm)	–2.9 (–42.32/22.6)	–5 (–17.9/–1)	–1.52 (–433/204)	0.423 ^u^

Difference (%)	–25.4 (–62.9/5.6)	–38.5 (–64.1/–10.6)	–16.0 (–58.5/62.8)	0.055 ^u^

**P-value (intragroup)**	0.198 ^w^	**0.013 ^w^**	0.989 ^w^	


TACE: Trans-arterial chemoembolization; DSMs: Degradable starch microspheres; DEB: Drug eluting beads ; AFP: alpha-fetoprotein; mRECIST: Modified Response Evaluation Criteria in Solid Tumors. ^u^ Mann Whitney U test(Monte Carlo), ^w^ Wilcoxon Sign Rank Test(Monte Carlo), ^ff^ Fisher Freeman Halton Test(Monte Carlo), Q1: 1th percentile, Q1: 3th percentile.

Baseline viable tumor diameters were similar between study groups, whereas DSMs-TACE vs. DEB-TACE was associated with significantly lower median (Q1-Q3) post-procedure viable tumor diameter (7 [0–15] mm vs. 18 [9–34] mm, p = 0.007), and significantly higher % reduction (–81.2 [–100.0– –66.7] % vs. –66.7 [–73.3 – –46.2] %, p = 0.005) of the viable tumor diameter (***Table 1***).

Change in total tumor diameter also revealed significantly higher reduction (median (Q1-Q3) –32.1 [–45.3 – –20.0] % vs. –13.3 [–30.0 – 0.0] %, p = 0.005) in the DSMs-TACE group (***Table 1***).

The decrease in median (Q1-Q3) serum AFP levels from baseline to post-procedure was significant only in the DSMs-TACE group (from 29.5 [6.8– 98.3] ng/mL to 11.4 [2.3 – 40] ng/mL, p = 0.013) (***Table 1***).

## Discussion

Our findings regarding efficacy of two different chemoembolization methods in a retrospective cohort of unresectable HCC patients revealed association of DSMs-TACE with higher treatment efficacy in terms of both initial (one month) biological and radiological tumor response when compared to DEB-TACE, but no significant difference was noted in terms of local recurrence or mortality during follow up.

Our findings support the consistently reported association of DSMs-TACE with favorable outcome in HCC patients in terms of treatment response based on mRECIST criteria [9,10,11,12]. The efficacy of DSMs-TACE was reported in a one-year follow-up study of 40 consecutive BCLC stage B or C patients (31 male; age, 70.6 ± 13.6 years) with intermediate or locally advanced HCC, including disease control rate of 52.5% with a median OS of 11.3 months [12]. In another study of 179 DSMs-TACE procedures in 50 patients with HCC, treatment outcome based on mRECIST revealed objective response rate of 44% and disease control rate of 70%, including CR, PR, SD and PD rates of 2%, 42%, 26% and 18%, respectively [13]. The treatment response based on mRECIST criteria in our patients treated with DSMs-TACE provided CR, PR, SD and PD rates of 27.6%, 65.5%, 6.9% and 0%, respectively; indicating an even more favorable response than in previous studies.

The transient short-term occlusion with rapid tissue reperfusion shortly after embolization obtained via DSMs-TACE has been considered likely to reduce hypoxia-dependent VEGF over production, and thus reduce the risk of rebound neovascularization, tumor re-growth and recurrence [5,9,10,11,12,13,14]. In the current study, survival and local recurrence rates were 26.1% and 31.6% in the DSMs-TACE group after median 36 months of follow up, while 14.3% and 16.7% in the DEB-TACE group after median 12 months of follow up. Although no significant advantage of DSMs-TACE was noted in terms of survival benefit or recurrence rate, it should be noted that there was a significant difference between groups in terms of follow-up length. Indeed, in studies with longer follow-ups, DEB-TACE has been associated with 3-year recurrence and survival rates of 65% and 26-29%, respectively [15].

Complete response and reduction in serum AFP levels were only observed in the DSMs-TACE group in our study, along with significantly better reduction in viable and total tumor diameters in the DSMs-TACE group after a median 1 (range 1 to 2) session. Indeed, given the higher rate of patients with BCLC-C advanced stage disease in the DSMs-TACE group at baseline, our findings tend to support that DSMs-TACE may allow extending treatment to patients with more severe liver disease via sparing of non-cancer parenchyma from ischemic injury [15].

In addition, DEB-TACE with smaller caliber beads (100–300 μm vs. 300–500 μm) has been considered to be more effective with higher chance of smaller particles to reach more distal locations without increasing complication rates [16,17,18,19]. Hence, given that our findings were related to comparison of 300–500 μm DEB with much smaller DSMs, the superior efficacy of DSMs-TACE over DEB-TACE may also be related to the difference in particle size and warrant further studies with comparable particle sizes.

The present study has a number of limitations including small sample size in each group, retrospective nature of study, significant differences in baseline characteristics of the two groups such as tumor stage, degree of tumor burden and AFP levels and the lack of analysis of toxicity. Lastly, our results may also be due to the differences among the two groups baseline characteristics, especially concerning the proportion of early-stage HCC (4% versus 27%, respectively for DSMs and DC Beads) as this probably affects the rates of CR.

## Conclusion

Both DSMs-TACE with 50 ± 7 *µm* microspheres 300-500 μm DEB-TACE are effective for local control for HCC, but our findings revealed superiority of the first method over the second in terms of initial (one month) biological (serum AFP levels) and radiological tumor response to treatment. Since we compared 300–500 μm DEB with much smaller DSMs in a small sample size with a short follow-up period, further prospective studies are needed to compare the outcomes of DSMs-TACE and DEB-TACE using comparable size particles.
